# Personalized Cutoffs for the Diagnosis of Neutropenic Fever Based on Patients' Baseline Body Temperature: A Retrospective Pilot Study

**DOI:** 10.7759/cureus.75163

**Published:** 2024-12-05

**Authors:** Ivayla I Geneva, Anthony J Corsi, Madison Searles, Christina D Lupone

**Affiliations:** 1 Internal Medicine, Crouse Hospital, Syracuse, USA; 2 Internal Medicine, Zucker School of Medicine at Hofstra/Northwell - North Shore University Hospital and Long Island Jewish Medical Center, New Hyde Park, USA; 3 Global Health and Translational Science, State University of New York Upstate Medical University, Syracuse, USA; 4 Public Health and Preventive Medicine, State University of New York Upstate Medical University, Syracuse, USA

**Keywords:** body temperature, length of stay, neutropenia, neutropenic fever, personalized medicine

## Abstract

Background

The management of neutropenic fever patients remains challenging. Patients' individual baseline body temperature may provide diagnostic and prognostic value.

Methods

This study is a retrospective analysis of 92 adults admitted for neutropenic fever to model the length of stay (LOS) and the ability to find a definitive diagnosis using the deviation of patients' temperature on admission from their outpatient baseline, acuity on admission, neutropenia level and persistence, fever persistence, and patients' age.

Results

Patients' average baseline body temperature was 36.7°C+/-0.3°C - the body temperature had to be over four standard deviations above the baseline to reach the gold standard fever threshold of 38.0°C. Their average fever on admission was 38.1°C. Fever etiologies were identified in 48%, and all constituted infections. Multiple regression modelling demonstrated that a longer LOS of >3 days was predicted by larger deviation from baseline body temperature at admission and by fever persistence at 72 hours post-admission, after correcting for the persistence of severe neutropenia (absolute neutrophil count <500) at 72 hours, age, neutropenia level, and need for intensive care unit admission. A similar model could not predict the ability to identify a fever-explaining diagnosis.

Conclusions

This pilot project provides support for the use of patients' individual baseline body temperature rather than a pre-established universal fever cutoff in the diagnosis of neutropenic fever. Using a personalized cutoff is expected to avoid missing cases. Further, deviation from patients' baseline body temperature at admission could serve as a predictor for the hospital LOS, which can serve as a potential tool for hospital bed management.

## Introduction

Human body temperature is a vital sign carefully monitored during all patient encounters, yet it remains controversial how each measurement should be interpreted [1-4]. Previously published analyses by our team demonstrated that both among healthy individuals [5] and hospitalized patients [6], there is a strong dependence of human body temperature on factors such as the individual's age and the measurement site. However, great variation was observed even among people in the same age group and using a single measurement site, with the standard deviations often exceeding 0.5°C [5]. Given this knowledge, researchers have recently suggested that "one size does not fit all" with regard to normal body temperature [7].

Understanding the predictive value of a given temperature measurement is of particular importance in the setting of the vulnerable patient population suffering from neutropenia, where it guides the initiation of antibiotics and hospitalization. Clinical practice guidelines, professional societies, and other authorities [8-10] define neutropenia as categories of absolute neutrophil count (ANC) as follows: mild neutropenia with ANC <1,500, moderate with ANC <1,000, severe with ANC <500, and profound with ANC <100, while the threshold for fever is defined as a single oral temperature of ≥ 38.3°C (101°F) or a temperature of ≥ 38.0°C (100.4°F) sustained over one hour [11]. Patients experiencing neutropenia, most commonly following chemotherapy, are accordingly instructed by their treating physician to seek medical attention if these temperature thresholds are reached so that outpatient broad-spectrum antibiotics can be started or the patient can be admitted to the hospital for intravenous antibiotics and further work-up. This would make sense if everybody's baseline body temperature was at 37°C -the agreed-upon "normothermia" level established more than a century ago [12], which had been recently challenged by several research groups [2, 13]. However, what if a patient's baseline body temperature is instead at 36°C - do we wait until it rises by a whole 2°C to reach 38°C before we call it "fever"? Conversely, if a patient's baseline body temperature runs at 37.5°C, do we judge a rise of 0.5°C, that reaches the 38°C conventional threshold to constitute a "fever"? After all, a 0.5°C interpersonal body temperature variation is expected to occur in health due to the diurnal variation driven by the human circadian rhythm alone [14]. To the best of our knowledge, no research has been carried out to address these important questions.

In this study, we examine the relationships between the change of body temperature from the baseline temperature of patients admitted for neutropenic fever and various characteristics of their hospitalizations, such as initial severity of the illness, neutropenia level of admission, fever persistence, neutropenia persistence, identification of infectious etiology for the fever, and the length of hospital stay. We use our findings to propose a new and personalized criteria for the definition of fever in neutropenic patients. Finally, we provide a literature review of relevant published studies.

This article was previously presented as a meeting abstract and poster presentation at the Annual Meeting of the Infectious Diseases Society of America in Washington, DC, in October 2022.

## Materials and methods

This retrospective analysis project was reviewed by the Institutional Review Board (IRB) of the State University of New York Upstate Medical University and was granted an exempt status under IRB # 1725508-1. All data were collected retrospectively from the electronic medical record database of our tertiary medical center. All patient information used in this study was completely de-identified. Patients aged >89 years of age were filed as >90 years old, following our IRB's guidelines.

We searched the database for patients with neutropenic fever who were admitted from December 1st, 2016, to December 1st, 2019, and met the inclusion and exclusion criteria as follows. The inclusion criteria were: 1) the patients had to be adults with age ≥18 years, 2) the admission diagnosis had to be "neutropenic fever", and 3) there existed outpatient baseline body temperature reading(s) within one year before admission. The exclusion criteria were: 1) there was a known infectious process before admission, 2) there was a documented use of current systemic antibiotics (including patients on prophylactic antibiotics) but EXCEPT for patients for whom antibiotics were started on the same day when they were sent to the hospital by, e.g., a medical provider in a clinic, 3) the absolute neutrophil count was ≥1500 cells/µL at the time of admission.

Several data categories were collected, either via automated data download routines or manually extracted from the charts: patient demographics (age and sex), baseline body temperature reported as the average from all outpatient body temperatures measured in the outpatient setting during routine outpatient clinic visits spanning the 12 months preceding the index admission, hospitalization-related information (primary service at time of admission, discharge location, whether the patient was alive at the time of discharge, length of stay, whether a specific diagnosis was identified during the 72 hours following admission that could account for the neutropenic fever, absolute neutrophil counts on admission and at later time periods up to 72 hours post-admission, body temperature as reported by the patient or their family that triggered the search for medical attention, maximal body temperature at time of admission (the maximum temperature recorded during the emergency department stint and the first 8 hours following the hospitalization), maximal body temperature from 25 to 48 hours and from 49 to 72 hours following the hospitalization). Measurement sites for the collected body temperatures were noted and subsequently standardized to oral using correction factors derived from a recent systematic review on normal body temperature [5]. Standardization to compensate for diurnal variations in patients' outpatient baseline body temperature was considered but ultimately judged to be inadvisable given the lack of reliable correction factors in health and disease and the previously published data showing how the body temperature diurnal variation is lost during hospitalization among both critically ill patients [15-18] and those not critically ill [14, 19]. Further, the baseline body temperature measurements took place in the clinics during the daytime, when the body temperature is expected to be mostly constant.

Descriptive statistics were used for patient demographics, admission to the intensive care unit (ICU) or to general floor unit, body temperature measurements, neutropenia levels where the ANC is measured in cells/µL (categorized into 1000 to 1500 (mild), 500 to <1000 (moderate), 100 to <500 (severe), and < 100 (profound)), length of stay (categorized into ≤3 days and >3 days), diagnosis accounting for the fever (categorized into urinary tract infections, upper respiratory tract infections, pneumonia, skin and soft tissues infections, gastroenterological infections, bacteremia, viremia, and discharge location. Further, it was also statistically inadvisable to treat the length of stay as a continuous variable due to the abundance of outliers for extended length of stay. The Exact Sign test was used to compare the self-reported maximal body temperature before admission and the maximal temperature measured on admission (the highest reading taken during the Emergency Department waiting time and the first 8 hours following admission). Linear regression was applied to evaluate the effect of the patient's age on the measured change in body temperature ΔT (maximal temperature measured on admission minus the personal baseline body temperature).

Multiple logistic regression with internal validation testing was used to model the length of stay based on age, neutropenia level on admission, the persistence of severe neutropenia (ANC <500) at 72 hours post-admission, ICU vs non-ICU level of care on admission, ΔT, the persistence of fever at 72 hours post-admission, and also controlling for use of scheduled acetaminophen, which is one of the most commonly used over-the-counter medication that may lower fevers. For the neutropenia categories, we had to combine mild and moderate neutropenia into a single category due to the low number of patients in those groups compared to both the severe and profound neutropenia groups. We created three models (A, B, C) using different cutoffs for what is considered fever. Namely, model A used a simple cutoff for all patients at body temperature ≥ 38°C. Model B used a personalized cutoff based on each patient's baseline body temperature and added to it two standard deviations (with 1 SD = 0.30°C based on all outpatient temperature measurements). Model C used a personalized cutoff at 3 SD above each patient's baseline body temperature. Similarly, three multiple logistic regression models (A, B, C) were created to predict the ability to identify a specific cause for the fever (e.g., a specific infectious process) based on age, neutropenia level on admission, persistence of severe neutropenia at 72 hours post-admission, ICU vs non-ICU level of care on admission, ΔT, persistence of fever at 72 hours post-admission, and the use of scheduled acetaminophen. Regarding missing data, by default, the SPSS routines did a listwise deletion of missing values. This meant that only cases with non-missing values for the dependent as well as all independent variables were used in the analysis.

## Results

Our database query for "neutropenic fever" as the admission diagnosis yielded a total of 316 admissions over a three-year period. Of those, we determined 92 admissions of 86 unique patients to have met all inclusion and exclusion criteria. Table 1 features patients' demographics and descriptive statistics associated with the included admissions. Both sexes were equally represented. Ages ranged from 20 to more than 90 years, with an average of 55 years. Only 11% of the admissions were directly to an intensive care unit (ICU). Most patients (49%) were admitted under hematology-oncology, with 14% admitted to General Medicine, 11% each to the bone marrow (BMT) unit and the Medical ICU, with the rest of the specialties comprising less than 5% of the admissions. The majority (55%) had profound neutropenia (absolute neutrophil count (ANC) <100), and 29% had severe neutropenia (100≥ ANC <500) at the time of admission. The reason for neutropenia in nearly all patients (95%) was chemotherapy treatment. A clear diagnosis to explain the neutropenic fever was identified in just under half of the admissions. All of the identified diagnoses were infections. The majority of the patients (80%) had been placed on scheduled Acetaminophen. Most admissions (89%) ended with discharge to home. Only two patients died during their hospital stay. The length of stay was at most three days for 44% and more than three days for 67% of patients, where the two deceased patients were not included. 

**Table 1:** Demographics and descriptive statistics The table features the demographics of the included patients as well as descriptive statistics regarding patients' acuity on admission, primary hospital service, neutropenia level on admission, the suspected reason for the neutropenia, whether the reason for fever was identified, and the etiology if applicable, information regarding the use of Tylenol, discharge location, length of stay, survival rate. SD - standard deviation; ICU - intensive care unit; BMT - bone marrow transplant; ANC - absolute neutrophil count measured in cells/µL; SLE - systemic lupus erythematosus.

Demographic category	# Patients in the category (percentage of total # patients)
Sex	
Female	47 (51.1%)
Male	45 (48.9%)
Age	
Mean +/- SD	54.9 +/- 17.9 years
Range	20 - more than 90 years
Acuity on admission	
ICU admission	10 (10.9%)
Non-ICU admission	82 (89.1%)
Primary service (top 4)	
Hematology-oncology	45 (48.9%)
General medicine	13 (14.1%)
BMT unit	10 (10.9%)
Medical ICU	10 (10.9%)
Cardiothoracic	4 (4.3%)
Pediatric service (patients ≥ 18 years old)	4 (4.3%)
Surgery transplant	3 (3.3%)
Neurosurgery	3 (3.3%)
Neutropenia level on admission	
Mild (ANC <1500), ANC in cells/µL	4 (4.3%)
Moderate (ANC <1000)	10 (10.9%)
Severe (ANC <500)	27 (29.3%)
Profound (ANC <100)	51 (55.4%)
Reason for neutropenia	
Chemotherapy	87 (94.6%)
Transplant	3 (3.3%)
SLE	1 (1.1%)
Unknown	1 (1.1%)
Reason for fever identified in 72 hours	
Yes	44 (47.8%)
No	48 (52.2%)
Specific diagnosis accounting for fever	
Pneumonia	10 (10.9%)
Bacteremia	9 (9.8%)
Gastroenterological infection	7 (7.6%)
Upper respiratory tract infection	5 (5.4%)
Skin and soft tissue infection	4 (4.4%)
Viremia	1 (1.1%)
Scheduled Tylenol use	
Yes	74 (80.4%)
No	18 (19.6%)
As needed Tylenol use	
Yes	59 (64.1%)
No	33 (35.9%)
Discharge location	
Deceased	2 (2.2%)
Home with self-care	55 (59.8%)
Home with home health care	27 (29.3%)
Skilled nursing facility	3 (3.3%)
Rehabilitation center	1 (1.1%)
Short term hospital	1 (1.1%)
Home with hospice	1 (1.1%)
Correction facility	1 (1.1%)
Left against medical advice	1 (1.1%)
Length of stay (days)	
≤3 days	40 (43.5%)
>3 days	50 (54.3%)
Not included (deceased)	2 (2.2%)
Survival	
Alive on discharge	90 (97.8%)
Deceased	2 (2.2%)

We analyzed patients' baseline body temperature and their body temperature readings during the hospitalizations with descriptive statistics as detailed in Table 2. We found that the average baseline body temperature was at 36.7 +/- 0.30°C, ranging from 35.8 to 37.5°C. We noted no outliers. The majority of the patients (60%) had more than one outpatient measurement, with an average outpatient measurements per person being five. The self-reported fever before admission to the hospital was at 38.72 +/- 0.45°C but the maximal temperature actually measured at the time of admission (the highest measurement during the ED stint and the first eight hours following hospitalization, combined) was significantly lower at 38.14 +/- 0.82°C, with p<0.001 using the Exact Sign Test for the 55 patients who had self-reported pre-hospitalization fevers recorded in the medical charts. The measured change from baseline body temperature at the time of admission, ΔT, which supposedly triggered the neutropenia fever admission, was on average 1.45 +/- 0.87°C (range: - 0.22 to + 3.81°C). Using linear regression, we found that ΔT was independent of patients' age (p=0.366), but there seemed to be a trend toward smaller ΔT as age increased. Based on the ≥ 38C cutoff, only 24% of patients had persistent fever over 48-72 hours, but based on personalized cutoffs at >2 standard deviations (SDs) or >3 SDs above their outpatient baseline, 54% and 34% had persistent fever, respectively. With regards to neutropenia persistence, which was defined as ANC <500 at 72 hours post-admission, there were 57 patients (72.2%) who featured persistence of neutropenia, while 22 patients (27.8%) did not; data on neutropenia level of 72 hours was not available for the remaining 13 patients. 

**Table 2:** Descriptive statistics of temperature readings The table features descriptive statistics regarding patients' baseline, i.e. outpatient, body temperatures as well as their body temperatures during the hospitalization. The table also provides information regarding patients’ level of neutropenia on admission to the hospital as well as during the hospitalization. All body temperatures had been standardized to oral. SD - standard deviation; ED - emergency department; ΔT - maximal temperature on admission minus outpatient baseline body temperature; ANC - absolute neutrophil count (cell count/μL).

Body temperature variable	Mean +/- SD, (Range)
Outpatient baseline body temperatures, standardized to oral, N=92	36.71 +/-0.30°C, (35.8-37.5)
Self-reported fever before admission, N=55	38.72 +/- 0.45°C, (37.83-40.00)
Maximal temperature on admission, standardized to oral, N=91	38.14 +/- 0.82°C, (36.87-39.70)
ΔT, N=90	1.45 +/- 0.87°C, (-0.22-3.81)
Maximal temperature 25-48 hours, N=87	37.60 +/- 0.79°C, (36.28-39.61)
Maximal temperature 49-72 hours, N=78	37.43 +/- 0.69°C, (36.61-39.61)
Persistence of Fever Variables	# Patients in the Category (Percentage of Total # Patients)
Persistence of fever (defined as T ≥ 38C), N=88	22 (23.9%)
Persistence of fever (defined as 2 SD above baseline), N=87	50 (54.3%)
Persistence of fever (defined as 3 SD above baseline), N=87	31 (33.7%)
Persistence of neutropenia (defined as ANC <500 at 72 hours), N=79	57 (72.2%)
Lack of persistence of neutropenia (ANC > 500 at 72 hours), N=79	22 (27.8%)

Our multiple logistic regression model (Table 3) demonstrated that a longer hospital length of stay (LOS) of >3 days was predicted by a larger deviation from baseline body temperature at admission and independently by fever persistence, after correcting for patients' age, neutropenia level on admission and at 72 hours, the need for ICU level of care on admission, and the use of scheduled Acetaminophen. Model A (using a uniform persistence of fever cutoff at ≥ 38°C) correctly classified the cases 81% of the time (where 70% minimum is needed for significance) and showed that every 1°C increase in ΔT is associated with an increase in the odds of being hospitalized for >3 days by 2.99-fold; also, that the persistence of fever increases these odds by 10.47-fold. Model B (using a personalized persistent fever cutoff at 2 SDs from each patient's pre-hospitalization body temperature baseline) correctly classified the cases 74% of the time and demonstrated that every 1°C increase in ΔT is associated with an increase in the odds of being hospitalized for >3 days by 2.28-fold. Finally, model C (using a personalized persistent fever cutoff at 3 SDs from each patient's pre-hospitalization body temperature baseline) correctly classified the cases 77% of the time and showed that every 1°C increase in ΔT is associated with an increase in the odds of being hospitalized for >3 days by 2.19-fold; also, that the persistence of fever increases these odds by 3.97-fold.

**Table 3:** Multiple logistic regression model for the hospital length of stay The table provides a summary of the statistics used to evaluate three models of the hospital length of stay. The models (A, B, and C) differ by the defined cutoff for fever, with the input variables being age, ANC on admission, ANC at 72 hours post-admission, acuity of illness on admission (ICU versus non-ICU level of care), the change in patients' temperature on admission from their baseline body temperature, the persistence of fever at 72 hours post-admission, and scheduled Tylenol use. ANC - absolute neutrophil count (cell count/μL); T - body temperature (degrees Celsius); ICU - intensive care unit; ΔT - difference in °C between baseline body temperature and the Tmax on admission; OR - odds ratio; CI - confidence interval; Bold indicates statistically significant results with p<0.05.

	Age		ANC on admission (<100 vs <500 vs 500 to 1500)		ANC at 72 hours (<500 vs ≥500)		ICU vs non-ICU level of care on admission		ΔT		Persistence of fever at 72 hours		Scheduled Acetaminophen use (yes/no)	
	p-value	OR, 95% CI, intercept	p-value	OR, 95% CI, intercept	p-value	OR, 95% CI, intercept	p-value	OR, 95% CI, intercept	p-value	OR, 95% CI, intercept	p-value	OR, 95% CI, intercept	p-value	OR, 95% CI, intercept
Model A - generic fever cutoff (Tmax ≥ 38.0°C)	0.249	1.02 (0.986-1.055) 0.020	0.379	3.12 (0.322-52.366) 1.139	0.922	1.10 (0.187-6.388) 0.088	0.999	1.32 (NA) 21.001	0.01	2.99 (1.290-6.508) 1.064	0.009	10.47 (1.779-61.634) 2.349	0.185	0.34 (0.062-1.707) -1.120
Model B - personalized fever cutoff (Tmax > 2SDs)	0.503	1.01 (0.980-1.043) 0.011	0.632	1.69 (0.223-19.418) 0.523	0.609	1.51 (0.309-7.392) 0.414	0.999	1.35 (NA) 21.023	0.023	2.28 (1.122-4.647) 0.826	0.497	1.54 (0.441-5.403) 0.434	0.547	0.61 (0.125-3.018) -0.489
Model C - personalized fever cutoff (Tmax > 3SDs)	0.405	1.03 (0.982-1.045) 0.013	0.588	2.10 (0.254-25.981) 0.742	0.708	1.40 (0.256-7.423) 0.322	0.999	1.28 (NA) 20.971	0.033	2.19 (1.066-4.487) 0.783	0.04	3.97 (1.067-14.759) 1.372	0.275	0.42 (0.086-2.012) -0.877

Multiple logistic regression models (Table 4) were used to study the ability to identify a specific cause for patients' fever (e.g., a specific infectious process) based on patients' age, neutropenia level on admission, persistence of severe neutropenia at 72 hours post-admission, ICU vs non-ICU level of care on admission, ΔT, persistence of fever at 72 hours post-admission (using the same cutoffs as described for models A, B, and C in the previous section), and the use of scheduled Acetaminophen. Unfortunately, none of the studied models yielded significant results with p<0.05.

**Table 4:** Multiple logistic regression model for identifying the fever etiology The table provides a summary of the statistics used to evaluate three models that aim to predict patients’ fever etiology. The models (A, B, and C) differ by the defined cutoff for fever, with the input variables being age, ANC on admission, ANC at 72 hours post-admission, acuity of illness on admission (ICU versus non-ICU level of care), the change in patients' temperature on admission from their baseline body temperature, the persistence of fever at 72 hours post-admission, and scheduled Tylenol use. ANC - absolute neutrophil count (cell count/μL); T - body temperature (degrees Celsius); ICU - intensive care unit; ΔT - difference in °C between baseline body temperature and the Tmax on admission; OR - odds ratio; CI - confidence interval

	Age		ANC on admission (<100 vs <500 vs 500 to 1,500)		ANC at 72 hours (<500 vs ≥500)		ICU vs non-ICU level of care		Δ T		Persistence of fever at 72 hours		Scheduled Acetaminophen use (yes/no)	
	p-value	OR, 95% CI, intercept	p-value	OR, 95% CI, intercept	p-value	OR, 95% CI, intercept	p-value	OR, 95% CI, intercept	p-value	OR, 95% CI, intercept	p-value	OR, 95% CI, intercept	p-value	OR, 95% CI, intercept
Model A - generic fever cutoff (Tmax ≥ 38.0°C)	0.477	1.01 (0.982-1.04) 0.010	0.555	0.55 (0.053-3.545) -1.026	0.989	1.01 (0.215-4.747) 0.011	0.261	2.80 (0.464-16.925) 1.031	0.689	1.13 (0.628-2.022) 0.120	0.347	1.78 (0.535-5.926) 0.577	0.457	1.77 (0.392-8.036) 0.573
Model B - personalized fever cutoff (Tmax > 2SDs)	0.672	1.01 (0.978-1.035) 0.006	0.558	0.35 (0.510-8.729) -1.052	0.942	1.06 (0.228-3.150) 0.057	0.272	2.73 (0.455-16.396) 1.005	0.768	1.09 (0.608-1.960) 0.088	0.527	1.45 (0.456-4.635) 0.374	0.459	1.81 (0.376-8.729) 0.594
Model C - personalized fever cutoff (Tmax > 3SDs)	0.572	1.01 (0.980-1.037) 0.008	0.493	0.32 (0.490-3.101) -1.130	0.935	1.11 (0.230-4.950) 0.064	0.25	2.86 (0.479-17.053) 1.050	0.757	1.11 (0.611-1.972) 0.093	0.696	1.24 (0.419-3.676) 0.216	0.376	2.00 (0.432-9.239) 0.692

## Discussion

The diagnostic uncertainty, the often-atypical presentations, and the need for empiric treatment render the management of patients with neutropenic fever challenging. We hypothesized that these patients' individual baseline body temperature provides diagnostic and prognostic value.

Our patients' average oral baseline body temperature was 36.7 +/- 0.3°C. This compares with the average oral temperature of 36.57°C reported in our recently published meta-analysis [5]. The intrapersonal variation is close to the previously reported variation of 0.32°C in healthy adults by Diamond et al. [7] and the 0.39°C to 0.4°C reported by Frazer et al. [20]. The fact that the self-reported Tmax prior to arrival in the Emergency Department was statistically higher than the measured Tmax on admission was most likely due to self-medication at home with Acetaminophen, for which, unfortunately, data was not available. Another important limitation in our dataset is the fact that, being a retrospective study, we did not have control over the collection of baseline body temperature in the outpatient clinic. This resulted in some patients having fewer measurements of baseline body temperature than others, this decreasing the accuracy. Yet, there were certain important trends we observed that can be further verified in larger and/or prospective studies.

Given the baseline body temperature of the studied patients, at two standard deviations (SDs) above their personal baseline, only 3% of our patients would be at the 38°C cutoff for fever, which is the current standard of care definition for fever; at 3 SDs, it would be only 20%. Therefore, it stands to reason that the standard 38°C cutoff is too high and would fail to identify many neutropenic fever cases. Further, recent studies [13] showed that there had been a steady decline in body temperature among humans over the last 150 years, at a rate of 0.03°C per birth decade. This again argues for the need to lower the cutoff for fever. But even more importantly, the great interpersonal variation between patients and healthy adults, which is on the order of over 1.5°C [5-7], clearly defies the usefulness of a universal fever cutoff. In many situations, particularly regarding immunosuppressed patients, we cannot afford to miss the development of fever, and it will be missed in a large portion of the cases if a universal fever cutoff is used. Instead, we suggest that each patient should have their baseline body temperature established via outpatient measurements in clinics and have it updated on a yearly basis since it is well-known that human body temperature decreases as we age [5]. Further, we propose that each patient should be instructed by their medical provider to look for a deviation from their personal baseline body temperature rather than making the decision to seek medical attention based on the standard 38°C cutoff. This is expected to prove useful in immunosuppressed patients like the ones included in our study but also in the general population, where fever is often the first indicator that an ailment such as cellulitis or pneumonia has led to systemic involvement requiring hospitalization. Further, we hope that our methodology will inform both clinicians and researchers to use standardization of body temperature, e.g. oral, in order to avoid undue body-site-specific temperature variations.

Further, our data demonstrated how the specific deviation from patients' baseline body temperature on admission could serve as a predictor for hospital length of stay (LOS) in patients with neutropenic fever. This correlation held true for fever cutoffs at 38°C (model A), as well as at 2 SDs (model B) or 3 SDs (model C) above patients' personal baseline body temperature. Of course, we must point out that a larger study sample and a prospective study design will likely be necessary to fine-tune the predictive value of fever here because fever is only one among many variables that influence the LOS. Nevertheless, we suspect that the association with LOS identified in our pilot study could help with hospital bed management.

Our next significant finding was that the persistence of fever among neutropenic patients was associated with longer LOS. In fact, the association was strongest for the 38°C cutoff (model A), which was not surprising as this is the default cutoff currently in use in healthcare. A larger study sample could make it possible to find out whether adopting the personalized cutoffs at 2 or 3 SDs above patients' baseline body temperature, which tends to be lower than the universal 38°C cutoff, would serve to prevent premature hospital discharge of neutropenic patients. We suspect this would be the case based on our recently published paper [21], where we demonstrated that the preventable readmissions among people with fever or hypothermia at the time of discharge were predominantly due to infections. In our current study of neutropenic fever patients, all the identified etiologies for the fever were infections. Using a personalized fever definition would aid the clinical decision-making with regard to hospital discharge.

Finally, we attempted to create a prediction model for the identification of neutropenic fever etiology, yet this yielded no significant results. This model was likely limited by the fact that less than 50% of included patients had a causative etiology identified, and this low percentage is comparable to those of previously published studies [22, 23]. The remainder of unidentified etiologies may have been secondary to inflammatory responses, adverse medication effects, or underlying malignancy.

Study limitations

Given the retrospective design of our study, the research findings will have to be validated in a prospective trial. Specifically, a retrospective design limits causal inference, particularly in linking baseline temperature deviations to length of stay--a problem that will be solved by a prospective study. Additional caveats for our work include the fact that the vast majority of the included patients were on scheduled acetaminophen and/or as-needed acetaminophen, which could have masked fevers during the hospitalization, thus introducing noise to our data. Further, the baseline body temperatures were based on only a handful of measurements per patient as these were collected retrospectively, thus increasing the chance for error. A potential confounder influencing the baseline body temperature was the time of the day when the measurement took place since body temperature fluctuates with circadian rhythmicity, which, unfortunately, we were not able to correct in this pilot study. Regarding missing data, e.g. not all patients had their neutrophil count measured at 72 hours, it remains possible that this can lead to selection bias in the patient population - a problem that could be addressed by a larger study. Also, we aimed to stratify the severity of illness of our patients by noting which ones were admitted directly to the ICU. Unfortunately, the ICU group was much smaller than the non-ICU group, thus rendering this variable less useful in the statistical analyses. Finally, it would have been beneficial to analyze the LOS using a cutoff of two days (48 hours), since this is the usual cutoff for observation status (less than 48 hours) versus inpatient status (more than 48 hours), and as such, it carries important implications for the hospital billing and insurance coverage. However, very few of the patients in our sample were discharged in under two days, thus making the two-day cutoff statistically inadvisable. A larger study may be able to address this issue in future research.

## Conclusions

In conclusion, neutropenic patients are at increased risk for developing life-threatening infections due to their immunosuppression. The first step in managing such patients requires recognition of the development of fever. Given the comparatively low baseline body temperature of 36.7°C+/-0.3°C seen in our dataset, using a personalized cutoff for fever such as two or three standard deviations above the baseline may provide a more accurate diagnosis than the gold standard cutoff of 38.0°C persistent fever or 38.3°C single measurement. Our study also demonstrates that the amount of deviation from the baseline body temperature at the time of admission could serve as a predictor of the hospital length of stay of neutropenic patients. The field would benefit from additional prospective studies to evaluate the usefulness of personalized body temperature cutoffs for fever in neutropenia as well as other patient populations.
